# Recent Changes in Patterns of Mammal Infection with Highly Pathogenic Avian Influenza A(H5N1) Virus Worldwide

**DOI:** 10.3201/eid3003.231098

**Published:** 2024-03

**Authors:** Pablo I. Plaza, Víctor Gamarra-Toledo, Juan Rodríguez Euguí, Sergio A. Lambertucci

**Affiliations:** Conservation Biology Research Group, Ecotone Laboratory, Institute of Biodiversity and Environmental Research (INIBIOMA), National University of Comahue–National Scientific and Technical Research Council, San Carlos de Bariloche, Argentina (P.I. Plaza, V. Gamarra-Toledo, S.A. Lambertucci);; Natural History Museum, National University of San Agustín de Arequipa, Arequipa, Peru (V. Gamarra-Toledo);; Ministry of Health of Tierra del Fuego, Ushuaia, Argentina (J. Rodríguez Euguí)

**Keywords:** avian influenza, emerging pathogens, highly pathogenic avian influenza, H5N1, mammals, public health, viruses, zoonoses

## Abstract

We reviewed information about mammals naturally infected by highly pathogenic avian influenza A virus subtype H5N1 during 2 periods: the current panzootic (2020–2023) and previous waves of infection (2003–2019). In the current panzootic, 26 countries have reported >48 mammal species infected by H5N1 virus; in some cases, the virus has affected thousands of individual animals. The geographic area and the number of species affected by the current event are considerably larger than in previous waves of infection. The most plausible source of mammal infection in both periods appears to be close contact with infected birds, including their ingestion. Some studies, especially in the current panzootic, suggest that mammal-to-mammal transmission might be responsible for some infections; some mutations found could help this avian pathogen replicate in mammals. H5N1 virus may be changing and adapting to infect mammals. Continuous surveillance is essential to mitigate the risk for a global pandemic.

Since last century, highly pathogenic avian influenza (HPAI) viruses have caused diverse waves of infection (*1*). However, the ongoing panzootic event (2020–2023) caused by HPAI A(H5N1) virus could become one of the most important in terms of economic losses, geographic areas affected, and numbers of species and individual animals infected (*1*–*4*). This pathogen appears to be emerging in several regions of the world (e.g., South America); it has caused death in domestic and wild birds but also in mammals (*2*,*5*,*6*). This trend is of great concern because it may indicate a change in the dynamics of this pathogen (i.e., an increase in their range of hosts and the severity of the disease) (*3*).

H5N1 has affected several mammal species since 2003 (*6*,*7*), thus raising concern because H5N1 mammalian adaptation could represent a risk not only for diverse wild mammals but also for human health (*8*–*10*). Unfortunately, information about this topic, especially related to the current panzootic (2020–2023), is disperse and available often only in gray literature (e.g., databases and official government websites). This fact complicates access and evaluation for many stakeholders working on the front lines (e.g., wildlife managers, conservationists, and public health authorities at regional and local levels).

For this article, we compiled and analyzed information from scientific literature about mammal species, including humans, naturally affected by the current panzootic event and compared those findings with the outcomes of previous waves of H5N1 infection. We focus particularly on the species infected, their habitat, phylogeny, and trophic level, and the sources of infection, virus mutations, clinical signs, and necropsy findings associated with this virus. We also address potential risks for biodiversity and human health.

## Methods

We compiled scientific information on mammals infected by H5N1 virus through October 2023. We considered only scientific information on mammal species infected naturally (i.e., experimental studies were not included). We performed 2 systematic searches in Scopus and Google Scholar, first using the terms “H5N1 AND mammal”; this search was divided into 2 periods (1996–2019 and 2020–2023) (Appendix Figure 1, 2). We then performed an additional search with no time restriction using the following key terms: “H5N1 OR HPAI OR Highly Pathogenic Avian Influenza AND mammal OR unusual host.” This additional search contributed no new articles on the study topic (Appendix Figure 3). We also adopted a snowball approach, examining all the references in the articles we found in our searches. We included review articles only if they contributed new information about mammal species infected naturally with H5N1; we excluded articles based on serologic surveys because of the difficulty in determining when infection occurred, which can introduce uncertainty into the diagnosis (*11*).

**Figure 1:**
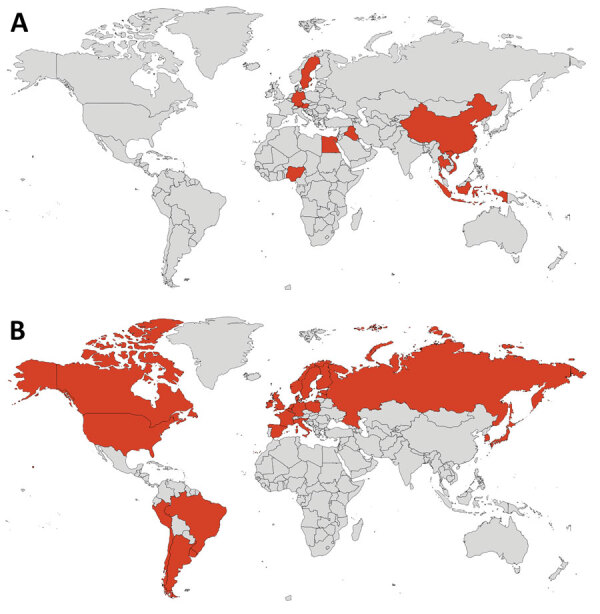
Geographic location of mammal species affected by highly pathogenic influenza virus A(H5N1) in previous waves of infection, 2003–2019 (A), and in the current panzootic, 2020–2023 (B).

**Figure 2:**
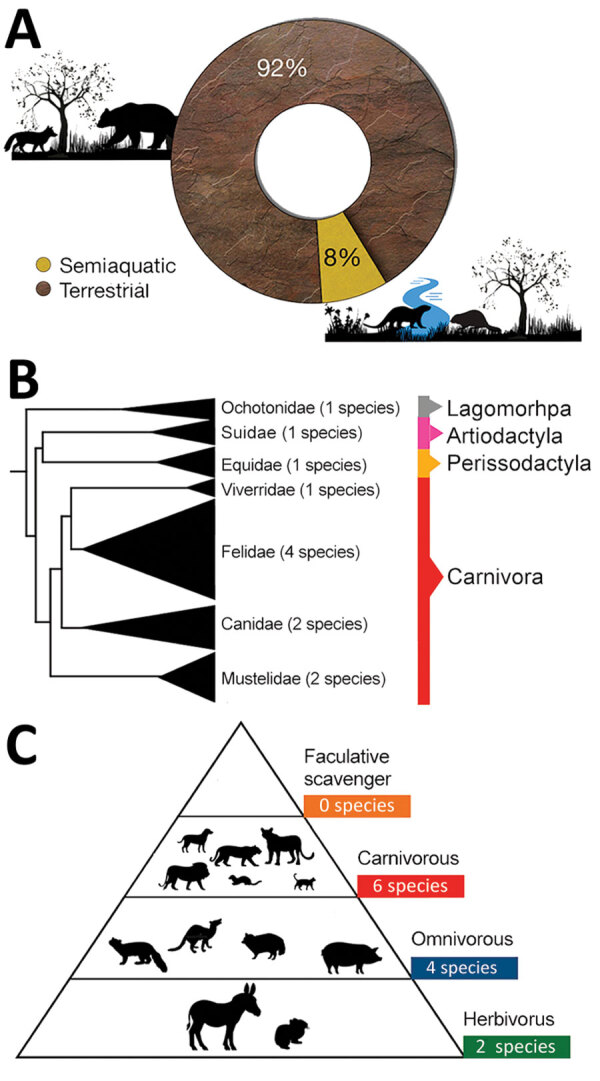
Characteristics of mammal species affected worldwide by highly pathogenic influenza virus A (H5N1) in previous waves of infection (2003–2019). A) Habitat of mammal species affected by H5N1. B) Phylogeny of mammal species affected (tree constructed using iTOL version 5 following Letunic and Bork [*15*], from DNA sequence data available in Upham et al. [*16*]). C) Trophic level (facultative scavenger, carnivore, omnivore, or herbivore) of mammalian species affected worldwide by H5N1.

**Figure 3:**
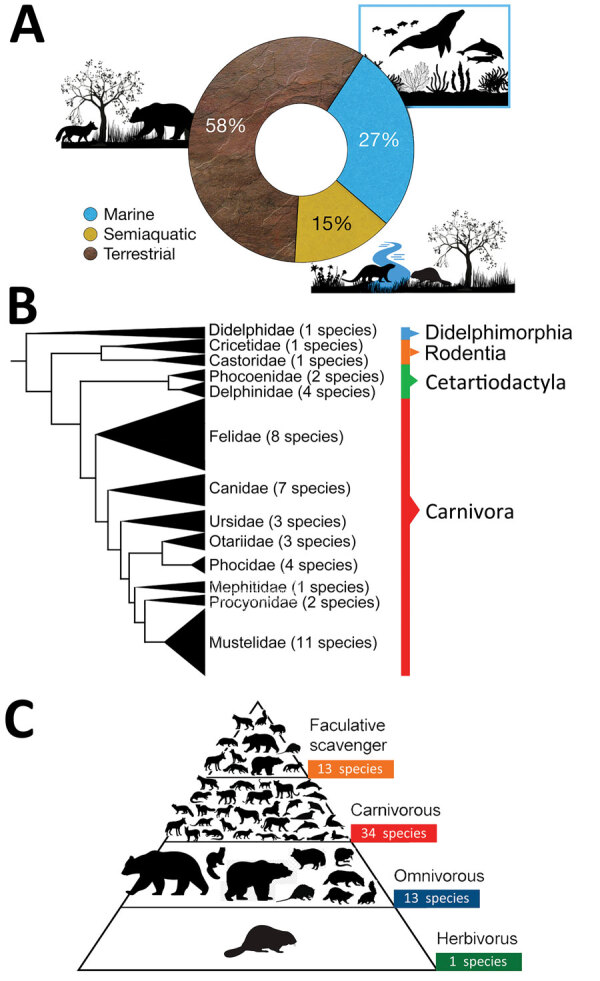
Characteristics of mammal species affected worldwide by highly pathogenic influenza virus A (H5N1) the current panzootic (2020–2023). A) Habitat of mammal species affected by H5N1. B) Phylogeny of mammal species affected (tree constructed using iTOL version 5 following Letunic and Bork [*15*], from DNA sequence data available in Upham et al. [*16*]). C) Trophic level (facultative scavenger, carnivore, omnivore, or herbivore) of mammal species affected worldwide by H5N1. Some of the omnivorous and carnivorous mammals included in the pyramid (n = 13) also consume carrion; thus, they are also considered to be facultative scavengers and are incorporated in the upper part of the pyramid.

To obtain additional information on the current panzootic event, we also searched the following official databases: World Organisation for Animal Health (*6*), the US Department of Agriculture’s Animal and Plant Health Service (*12*), and the United Kingdom’s Animal and Plant Health Agency (*13*). To obtain information about humans affected by this pathogen we used information provided by the World Health Organization (*14*). We constructed a map with the countries with reports of mammal infections (Figure 1) and the phylogeny of mammal species affected by H5N1 (Figures 2, 3) by using iTOL version 5, following Letunic and Bork (*15*), from DNA sequence data available in Upham et al. (*16*). We retrieved the conservation statuses of infected mammals from International Union for Conservation of Nature Red List of Threatened Species (*17*) and information on their diets from that database and MammalBase (*18*).

## Results and Discussion

### Scientific Information Available

We found 59 scientific articles on mammals infected naturally by H5N1 virus, 23 from previous waves of infection (up to 2019) and 36 from the current panzootic event (Appendix Figure 1, 2). The articles reporting mammals infected naturally in previous waves were published during 2004–2018, whereas those addressing the current panzootic were published during 2021–2023. The current panzootic has thus generated more articles in 3 years than all the previous waves of infection (published over a 15-year period). This fact suggests increased general interest in emerging pathogens affecting biodiversity and mammals (wild and farmed) and also that the current panzootic event is causing greater concern and having a greater effect than previous ones (considering the geographic regions and mammal species affected) (*4*).

### Geographic Localization of Information and Mammal Species Affected

During previous waves of infection, 10 countries reported mammals (not including humans) naturally infected by H5N1 (5 countries in Asia, 3 in Europe, and 2 in Africa) (Figure 1, panel A; Appendix Table). In the current event, 26 countries have reported information on mammals (not including humans) infected by this virus; most information is from Europe (17 countries), followed by South America (5 countries), North America (2 countries), and Asia (2 countries) (Figure 1, panel B; Appendix Table). To the best of our knowledge, for the current outbreak, no information is available on mammals from other parts of the world, which can probably be explained by a lack of testing or reporting of cases. Our review suggests that H5N1 virus is expanding its geographic range to new continents such as North and South America (Figure 1). This fact is of concern because when an emerging pathogen reaches naive populations, the consequences for biodiversity can be catastrophic, especially for threatened species (*19*).

We found that previous waves of infection affected several mammals around the world (*7*,*20*); for example, tigers (*Panthera tigris*), leopards (*Panthera pardus*), domestic cats (*Felis catus*), domestic dogs (*Canis lupus familiaris*), Owston’s palm civet (*Chrotogale owstoni*), stone martens (*Martes foina*), plateau pikas (*Ochotona curzoniae*), minks (*Neovison vison*), and raccoon dogs (*Nyctereutes procyonoides*) (Appendix Table). All the mammal species affected were terrestrial or semiaquatic species (Figure 2, panel A). Most mammals infected during previous waves (75%; n = 9) belong to the order Carnivora, whereas the remainder correspond to the Lagomorpha, Artiodactyla, and Perissodactyla orders (Figure 2, panel B). Infected mammal species included top predators (e.g., tigers and leopards) and some mesopredators (e.g., minks) (Appendix Table). Most species infected in previous waves were carnivores (n = 6) and omnivores (n = 4), followed by herbivores (n = 2) (Figure 2, panel C; Appendix Table).

So far, in the current panzootic, >48 mammal species from disparate regions of the world have been reported as naturally infected by H5N1 (Appendix Table). Most of those species (n = 35) are terrestrial or semiaquatic mammals (Figure 3, panel A; Appendix Table), but 13 species of marine mammals also were affected, resulting in massive deaths (up to thousands of individual animals) in geographic regions such as Peru, Chile, and Argentina (Figure 3, panel A; Appendix Table). Of the total number of mammals infected, 81% (n = 39) belong to the order Carnivora, and the remainder correspond to Didelphimorphia, Rodentia, and Cetartiodactyla (Figure 3, panel B). Infected mammal species include top predators (e.g., mountain lion [*Puma concolor*]) and several mesopredators (e.g., red fox [*Vulpes vulpes*]) (Appendix Table). Most mammal species infected are carnivores (n = 34), followed by omnivores (n = 13) and herbivores (n = 1); some of those species (n = 13) also are considered facultative scavengers (i.e., they include in their diet a considerable quantity of carrion; in our case to be a facultative scavenger carrion should be named in the diet) (Figure 3, panel C; Appendix Table).

The species infected in the 2 events show similarities. Most species belong to the order Carnivora and are top or mesopredators with a carnivorous diet; some species also are facultative scavengers. However, in the current panzootic event, the diverse marine mammals affected have suffered massive deaths (e.g., American sea lion [*Otaria flavescens*]) (Appendix Table). Marine mammals have been affected by other influenza viruses such as H10N7 (*21*), but the species affected and the number of dead individual animals attributable to the current event is of great concern (*22*,*23*); for example, the proportion of American sea lions that died in Peru represents 5% of their population there (*22*).

The current panzootic is ongoing, and the number of species being infected naturally is increasing (40 new mammal species have been reported as infected by this pathogen during the current panzootic), so the effect on mammal species may continue to worsen with time. This effect could just be attributable to the current high H5N1 infection rates throughout the world, which means the virus is reaching more areas and mammal species living in these places (i.e., high environmental circulation of this pathogen) (*8*). However, the dynamics of the virus may also be changing (*3*), in which case its infectivity in unusual species such as mammals is probably increasing (*8*). During the final review process of this article, 2 additional species were reported to be infected by this virus in the United States: the Abert’s squirrel (*Sciurus aberti*) and the polar bear (*Ursus maritimus*) (newly infected species are not shown in figures or the Appendix Table) (*6*).

### Source of Infection

Although the source of infection in mammals is often unknown, most scientific information available during previous and the current H5N1 event suggests that the most plausible source of infection is close contact with infected birds, including their ingestion, which may occur through predation of sick individual animals or scavenging on carcasses. For instance, in the year 2004, a total of 147 tigers and 2 leopards housed in zoos in Thailand became infected and died after consuming infected chicken carcasses (*24*,*25*). In China, this infection source was also associated with the death of a tiger in 2013 (*26*) and a lion in 2016 (*27*). In the current panzootic, the first case of H5N1 infection in minks in Spain was probably caused by contact with infected birds (perhaps gulls) (*9*). Ingestion of infected bird carcasses was probably the route of infection of red foxes in the Netherlands, Finland, and Japan during 2020–2022 (*28*–*31*), American sea lions in Peru in 2023 (*22*), diverse mesocarnivores in Canada during 2021–2022 (*32*) and otters (*Lutra lutra*) and a lynx (*Lynx lynx*) in Finland in 2021–2022 (*31*). Of concern, studies in infected tigers, farmed minks, and social species such as American sea lions, raise an alarm that mammal-to-mammal transmission may have occurred (*9*,*22*,*24*,*33*), but further research is needed to confirm this possibility.

If mammal-to-mammal transmission occurs during the current H5N1 panzootic, such transmission could imply that the virus mutated to enable virus replication in mammal tissues (*9*). Some researchers have reported mutations compatible with adaptation to mammal replication (*9**,**25**,**33**,**34*), which is concerning and requires attention. However, evaluating whether those mutations happen in wild birds before mammal infections or arise de novo in mammals after infection is important.

### Mutations Found

Through sequencing of the H5N1 viruses infecting mammals, some relevant mutations such as E627K in polymerase basic protein 2 (PB2) (PB2-E627K) and D701N in polymerase basic protein 2 (PB2) (PB2-D701N) have been found in previous waves and in the current panzootic (Appendix Table). Those mutations are commonly associated with virulence and efficiency in the replication of this pathogen in mammals (*31*,*33*,*35*). For instance, during 2004–2005, in Thailand, the isolated H5N1 viruses that infected tigers, a domestic cat, a domestic dog, and a leopard contained the PB2-E627K mutation (*25*,*35*,*36*). In the current panzootic, red foxes from the Netherlands also showed the mammalian adaptation of PB2-E627K (*28*). In viruses collected from red foxes, an otter, and a lynx in Finland in 2021–2022, the PB2-E627K and PB2-D701N mutations were identified (the latter mutation was reported in 1 red fox and 1 lynx in Finland) (*31*). Similarly, in the current panzootic, red foxes, otters, and polecats (*Mustela putorius*) in the Netherlands, and red foxes in Canada, and the United States had the PB2-E627K mutation (*8*,*32*,*37*). The PB2-E627K and PB2-D701N mutations were also detected in harbor seals (*Phoca vitulina*) in the United States (*34*), and the latter mutation was found in South American sea lions in Peru (*33*), and in a red fox in Canada (*32*). In both previous and current events, other mutations meriting further research were also found in diverse mammal species, including terrestrial, semiaquatic, and marine mammals (Appendix Table).

Mutations that facilitate replication of the virus in mammal hosts (e.g., enhancing polymerase activity in mammal cells), such as PB2-E627K and PB2-D701N, could be of concern (*8*,*31*,*33*). Potential mutations must be continuously scrutinized to detect whether the H5N1 virus is adapting to mammal-to-mammal transmission. This approach is important for wildlife conservation because if such transmission occurs, the consequences for threatened mammal species could be severe (e.g., threatened South American sea lion deaths in Peru [*22*]). In addition, mutations must be monitored for changes that may favor transmission to and between humans, which would increase the risk for a pandemic.

### Clinical Signs of H5N1 in Mammals

The most common clinical signs reported in infected mammals, both in previous waves and the current H5N1 panzootic, are neurologic and respiratory. For instance, in 2005, an infected Owston’s civet in Vietnam showed loss of appetite and neurologic signs such as convulsions and paralysis; the same clinical signs were reported in a stone marten in Germany in 2006 (*38*,*39*). Similarly, hundreds of infected tigers in a zoo in Thailand showed respiratory and neurologic signs before they died (*24*). In the current panzootic event, infected minks from Spain manifested loss of appetite, hyper salivation, depression, bloody snout, and neurologic signs such as ataxia and tremors (*9*). American sea lions in Peru and harbor seals in the United States showed respiratory signs (dyspnea and whitish secretions in nares) and neurologic signs (tremors and convulsions) (*22*,*34*). Red foxes, an otter, a polecat, and a badger (*Meles meles*) in the Netherlands had neurologic signs such as convulsions and head shaking (*8*,*30*). In Finland, an infected otter was also reported to have a set of neurologic signs (*31*). Finally, in the United States and Canada, several mammals manifested neurologic and respiratory signs (*32*,*37*). Those findings suggest that H5N1 virus has neurotropism in mammals, as reported in birds (*6*,*28*), causing severe disease and pathologic lesions (e.g., encephalitis); brain samples should be included in wildlife surveillance programs for reliable detection of the H5N1 virus in mammals (*8*).

Although neurologic and respiratory signs are commonly reported in mammals infected with H5N1, some species and individual animals show subclinical disease. For instance, infected pigs (*Sus scrofa domesticus*) from Indonesia, Nigeria, and China had no signs of influenza but tested positive for H5N1 (*40*–*42*). Similarly, in Austria, infected domestic cats display asymptomatic infections (*43*). Subclinical infections are concerning because they are not easily detected; infected individual animals may be transmitting the virus to other species and even humans, representing a risk to the ecosystem and human health (*40*,*41*).

### Necropsy Findings

In previous waves of infection and the current H5N1 panzootic, the most frequently reported anatomopathologic lesions in infected mammals were pneumonia and encephalitis. Those kinds of lesions (e.g., congestion of brain, meningoencephalitis, hemorrhagic lungs, and pleural effusion) were reported in dead tigers in Thailand and China during 2004–2014 (*24*,*26*,*44*), in a lion in China in 2016 (*27*), and in cats and dogs infected naturally in Thailand in 2004 (*45*,*46*). In the current panzootic, for instance, red foxes from the Netherlands had collapsed lungs with a marbled red aspect; histopathologic analyses showed a subacute to chronic purulent granulomatous broncho-interstitial pneumonia and nonsuppurative encephalitis with perivascular cuffing (*28*). Red foxes, polecats, otters, and a badger in the Netherlands also showed nonsuppurative meningitis, encephalitis, or meningoencephalitis, all with differences in severity (*8*). American sea lions in Peru had congestive brains compatible with encephalitis (*22*). A porpoise (*Phocoena phocoena*) in Sweden manifested meningoencephalitis (*47*). Similar findings, meningoencephalitis and pneumonia, were also found in mammals in Finland, the United States, and Canada (*31*,*32*,*37*).

Those findings suggest that respiratory and neurologic lesions are the most common pathologies of necropsied mammals infected with H5N1 in both previous waves of infection and the current panzootic. The lesions largely explain the neurologic and respiratory signs observed in mammals affected by this virus. Complete necropsies of infected mammals may help determine whether those anatomopathologic findings are frequent and pathognomonic for this disease in every species and most individual animals, as preliminary results suggest.

### Risks for Biodiversity

The current panzootic is affecting a larger number of species around the world than previous waves of H5N1 infection, and some are of conservation concern. Previous waves affected 2 endangered and 2 vulnerable species (Appendix Table). The current panzootic has so far affected 4 near threatened, 4 endangered, 3 vulnerable, and 1 critically endangered species (Appendix Table); this emerging pathogen may affect species of conservation concern, exacerbating their situation.

In general, most mortality events associated with the current panzootic appear to affect few individual animals and in only certain areas; thus far, large populations have not been affected in the way wild birds have been affected (*4*,*6*). However, this virus is suspected of producing massive deaths in some marine mammals; for example, >20,000 South American sea lions were reported to have died suddenly, and many individual animals tested positive for H5N1 (*6*,*22*,*23*). This fact raises concern as to the potential effect of this virus on the demography of some threatened mammal populations. This emerging pathogen represents a new species invading and impacting new environments and species and could therefore constitute a new threat for diverse species currently threatened by human action (e.g., land use change, contamination, and habitat loss) (*19*,*48*).

### Potential Risks for Human Health

During 2003–2023, a total of 878 humans tested positive for the H5N1 virus, and 458 deaths were reported, indicating a lethality of ≈52% (*14*). During 2003–2019, most human cases came from Asia and Africa, particularly from China (n = 53), Egypt (n = 359), and Indonesia (n = 200). From 2020 through July 2023, human cases of H5N1 infection occurred in diverse countries, such as Laos (1 case), India (1 case), United Kingdom (4 cases), China (2 cases), the United States (1 case), Vietnam (1 case), Spain (2 cases), Ecuador (1 case), Chile (1 case), and Cambodia (2 cases) (*14*). Those recent cases resulted in >3 deaths (*14*). Of note, this zoonotic virus has produced human cases in new geographic areas, such as South America.

The spillover to humans has been associated with close contact between humans and infected animals, particularly poultry; this kind of contact is relatively common in some geographic regions (even close contact between dead mammals and humans, as in Peru [*22*]). So far, no evidence indicates human-to-human transmission, and the risk for a pandemic event still seems low (*8*). However, one of the most severe influenza viruses to have affected humans (i.e., Spanish influenza [1918–1919]) developed from an avian influenza virus that adapted to humans (*49*), a fact that should be considered when assessing the spillover risk.

Mutations in the virus found in diverse mammal species, especially in the current panzootic, are of great concern. For instance, the T271A mutation reported in minks in Spain is also present in the H1N1 that produced a pandemic in 2009 (*9*). Similarly, the PB2-E627K mutation found in this virus in diverse geographic areas could indicate an adaptation for replication in mammals (*28*,*31*). Moreover, some infected species, such as minks, may act as a mixing vessel for interspecies transmission between birds, mammals, and humans (*9*). Mutations and infections with H5N1 in potential mixing-vessel species (e.g., minks and wild and domestic pigs) should be followed closely because of the potential risk to human health.

## Final Considerations

Given the magnitude of the current H5N1 panzootic, continuous surveillance is necessary to identify any increase in risk to biodiversity and human health. It is therefore essential that all affected countries share all their available information (e.g., genomic data of the H5N1 virus, species, and number of individual animals affected). We urge that all findings be shared quickly. International collaboration must be intensified to obtain rapid results; some less-developed regions have technologic and logistic barriers that hinder the production and analysis of information on the impact of this virus, and they may need help. There is a need for strong collaborative work between countries and institutions in preparation for any spillover that may lead to a mammalian panzootic or human pandemic.

It is fundamental that we rethink the interface between humans, domestic animals, and wild animals to prevent the emergence of dangerous pathogens that affect biodiversity and human health (*48*). Governments must assume responsibility for protecting biodiversity and human health from diseases caused by human activities, particularly diseases originating from intensive production (*50*), such as this H5N1 avian influenza virus. If we hope to conserve biodiversity and protect human health, we must change the way we produce our food (poultry farming, in this specific case) and how we interact with and affect wildlife.

Appendix Additional information about recent changes in patterns of mammal infection with highly pathogenic avian influenza A(H5N1) virus worldwide.
