# Supplementary material for: Recent Changes in Patterns of Mammal Infection with Highly Pathogenic Avian Influenza A(H5N1) Virus Worldwide
Source: Emerg Infect Dis. 2024 Mar;30(3):444–52. doi: 10.3201/eid3003.231098 (PMC10902543; doi:10.3201/eid3003.231098)
Supplement: Appendix — Additional information about recent changes in patterns of mammal infection with highly pathogenic avian influenza A(H5N1) virus worldwide. [file 23-1098-Techapp-s1.pdf]

Article DOI: https://doi.org/10.3201/eid3003.231098

Recent Changes in Patterns of Mammal Infection with Highly Pathogenic Avian Influenza A(H5N1) Virus Worldwide

Appendix

Appendix Table. Mammals infected by H5N1 or H5NX in previous infection waves and current panzootic, conservation status, order, family, diet consumed, number of individuals affected, mutations found and country of origin

Order	Family	Species	Conservation status**	Diet***	Individuals affected	Mutations found****	Country	References
PREVIOUS INFECTION WAVES (2003–2019)								
Terrestrial and semi-aquatic mammals								
Carnivora	Canidae	Raccoon dog (*Nyctereutes procyonoides*)	LC	O	At least 2	T/S156A, PB1-P13, PB1-Y436	China	(*1*)
	Canidae	Dog (*Canis lupus familiaris*)	NC	C	At least 1	PB2-E627K, M2-Ser31Asn, M2-Leu26I1e	Thailand	(*2*,*3*)
	Mustelidae	Mink (*Neovison vison*)	LC	C	At least 371	M2-S31N, NS1-G149A, NS1-D92E, HA-E513G	China, Sweden	(*4*–*6*)
	Mustelidae	Stone marten (*Martes foina*)	LC	O	At least 1	—	Germany	(*7*)
	Viverridae	Owston's civet (*Chrotogale owstoni*)	EN	O	At least 3	—	Vietnam	(*8*)
	Felidae	Tiger (*Panthera tigris*)	EN	C	At least 150	PB2-E627K, 31(N; Asparagine), HA-Asp94Asn, HA-Ser133Ala, HA-Thr156Ala, HA-Thr188Ile, HA-Lys189Arg, NA-His254Tyr, PB2-Asp701Asn, M1-Asn30Asp, M1-Thr215Ala, M2-Ser31Asn, NS1-Leu98Phe, NS1-Ile101Met, HA-Ser155Asn, HA-Gln222Leu, HA-Gly224Ser, PB2-Lys627Glu, NS1-Pro42Ser, NS1-Asp87Glu	Thailand, China	(*9*–*13*)
	Felidae	Lion (*Panthera leo*)	VU	C	At least 1	—	China	(*14*)

Order	Family	Species	Conservation status**	Diet***	Individuals affected	Mutations found****	Country	References
	Felidae	Leopard (*Panthera pardus*)	VU	C	At least 2	PB2-E627K	Thailand	(*11*)
	Felidae	Cat (*Felis catus*)	NC	C	At least 13	PB2-E627K, M2-Ser31Asn, M2-Leu26I1e	Austria, Thailand, Germany, Iraq	(*2*,*15*–*18*)
Lagomorpha	Ochotonidae	Plateau pikas (*Ochotona curzoniae*)	LC	H	At least 5	—	China	(*19*)
Artiodactyla	Suidae	Pigs (*Sus scrofa domesticus*)	NC	O	At least 70	Ala134Ser-viral receptor	China, Nigeria, Indonesia	(*20*–*22*)
Perissodactyla	Equidae	Donkey (*Equus asinus*)	NC	H	At least 3	HA-98(Y to N), HA-193(K to R), HA-216(E to K), HA-221(P to S)	Egypt	(*23*)
CURRENT PANZOOTIC (2020–2023)								
Terrestrial and semi-aquatic mammals								
Carnivora	Canidae	Red fox (*Vulpes vulpes*)	LC	C/FS	At least 290	PB2-E627K, NP-G485R, PB2-A152T, PB2-T521I, PB1-M644V, NP-A336T, NA-L22S, NS-D209N, PB2-D701N, HA-S137A, HA-T160A, HA-A185E, HA-D195T, HA-V198I, HA-E268G, HA-V210A, PB2-V292I, PB1-D622G, PB1-N375S, PA-P28L, PA-A36V, HA-N101S, HA-V214A, NA-A395E, NA-S248N, NA-H155Y, NA-N366S, M1-N30D, M1-T215A, M2-S82N, NS1-V2261, NS1- P42S, NS1-I106M	Netherlands, Sweden, Finland, Estonia, Ireland, Norway, Belgium, UK, France, Italy, Japan, Germany, USA, Canada, Latvia, Denmark,	(*24*–*46*)
	Canidae	Arctic fox (*Vulpes lagopus*)	LC	C/FS	At least 2048	—	Finland	(*27*,*43*,*45*)
	Canidae	Grey fox (*Urocyon cinereoargenteus*)	LC	C/FS	At least 1	—	USA	(*36*)
	Canidae	Raccoon dog (*Nyctereutes procyonoides*)	LC	C	At least 5	—	Japan, Finland	(*27*,*28*,*33*,*37*,*39*–*41*,*43*–*45*)
	Canidae	South American bush dog (*Speothos venaticus venaticus*)	NT	C	At least 1	—	UK	(*41*,*44*,*45*)
	Canidae	Coyote (*Canis latrans*)	LC	C/FS	At least 1	—	USA	(*24*,*27*,*28*,*30*,*36*,*37*,*40*,*41*,*44*,*45*)
	Canidae	Dog (*Canis lupus familiaris*)	NC	C	At least 1	—	Canada	(*27*,*41*,*44*,*45*)

Order	Family	Species	Conservation status**	Diet***	Individuals affected	Mutations found****	Country	References
	Mustelidae	Otter (*Lutra lutra*)	NT	C	At least 7	PB2-E627K	Finland, Netherlands, UK	(*25–29*,*31*,*37*,*39–41*,*44–46*)
	Mustelidae	Polecat (*Mustela putorius*)	LC	C	At least 7	PB2-E627K, PB2-T271A	Netherlands, Slovenia, Belgium	(*26–29*,*37–41*,*44*,*45*)
	Mustelidae	Badger (*Meles meles*)	LC	O	At least 1	—	Netherlands	(*28*,*29*,*37*,*38*,*40*,*41*,*44*,*45*)
	Mustelidae	Fisher cat (*Pekania pennanti*)	LC	O/FS	At least 4	—	USA, Canada	(*24*,*27*,*28*,*30*,*36*,*37*,*40*,*41*,*44*,*45*)
	Mustelidae	Mink (*Neovison vison*)	LC	C	>50000	HA-A185E, HA-D195T, HA-V198I, HA-E268G, HA-V210A, HA-S137A, HA-T160, PB2-T271A, PB1-388R, PB1-317V, PB1-F2–30L, NA-74S, NS2-13G, PA-56T, NA-163L	Canada, Spain, Finland	(*24*,*27*,*28*,*35*,*37*,*40*,*41*,*43–45*,*47*)
	Mustelidae	Stone marten (*Martes foina*)	LC	O	At least 1	—	Netherlands	(*41*,*44*,*45*)
	Mustelidae	Pine marten (*Martes martes*)	LC	O/FS	At least 1	—	Germany	(*27*,*45*)
	Mustelidae	Marine otter (*Lontra felina*)	EN	C	At least 2	—	Chile	(*27*,*41*,*44*,*45*; Pardo Roa et al.†)
	Mustelidae	Huillin (*Lontra provocax*)*	EN	C	At least 1	—	Chile	(*27*,*44*,*45*)
	Mustelidae	American marten (*Martes americana*)	LC	O/FS	At least 1	—	USA	(*27*,*30*)
	Mustelidae	North American river otter (*Lontra canadensis*)	LC	C	At least 2	—	USA, Canada	(*24*,*27*,*30*,*41*,*44*,*45*)
	Procyonidae	Raccoon (*Procyon lotor*)	LC	O	At least 18	PB2-E627K	USA, Canada	(*24*,*27*,*28*,*30*,*36*,*37*,*40*,*41*,*44*,*45*)
	Procyonidae	South American coati (*Nasua nasua*)	LC	O	At least 19	—	Uruguay, Germany	(*27*,*44*,*45*)
	Felidae	Bobcat (*Lynx rufus*)	LC	C/FS	At least 6	—	USA	(*24*,*27*,*28*,*30*,*36*,*37*,*40*,*41*,*44*,*45*)
	Felidae	Lynx (*Lynx lynx*)	LC	C	At least 1	PB2-E627K, PB2-D701N	Finland	(*26*,*28*,*37*,*39–41*,*44–46*)
	Felidae	Tiger (*Panthera tigris*)	EN	C	At least 1	—	USA	(*24*,*27*,*28*,*30*,*41*,*44*,*45*)

Order	Family	Species	Conservation status**	Diet***	Individuals affected	Mutations found****	Country	References
	Felidae	Mountain lion (*Puma concolor*)	LC	C	At least 21	—	USA	(*24*,*27*,*28*,*30*,*41*,*44*,*45*)
	Felidae	Lion (*Panthera leo*)*	VU	C	At least 1	—	Peru	(*27*,*45*)
	Felidae	Caracal (*Caracal caracal*)	LC	C	At least 1	—	Poland	(*44*,*45*)
	Felidae	Cat (*Felis catus*)	NC	C	At least 35	PB2-E627K, NS2-E26G, PB2-K526R	USA, France, Canada, Korea (Republic of), Poland	(*27*,*28*,*41*,*44*,*45*,*48*–*52*)
	Felidae	Leopard (*Panthera pardus orientalis*)	CE	C	At least 1	---	USA	(*24*,*27*,*28*,*30*,*40*,*41*,*44*,*45*)
	Mephitidae	Skunks (*Mephitis mephitis*)	LC	O/FS	At least 90	HA-S137A, HA-T160A, HA-A185E, HA-D195T, HA-V198I, HA-E268G, HA-V210A	USA, Canada	(*24*,*27*,*28*,*30*,*35*–*37*,*39*–*41*,*44*,*45*)
	Ursidae	Asiatic black bear (*Ursus thibetanus*)	VU	O/FS	At least 1	—	France	(*41*,*44*,*45*)
	Ursidae	American black bear (*Ursus americanus*)	LC	O/FS	At least 7	PB2-D701N	USA, Canada	(*24*,*27*,*28*,*30*,*37*,*40*,*41*,*44*,*45*,*53*)
	Ursidae	Grizzly bear (*Ursus arctos*)	LC	O/FS	At least 4	—	USA	(*24*,*27*,*28*,*30*,*40*,*41*,*44*,*45*,*54*)
Didelphimorphia	Didelphidae	Virginia opossum (*Didelphis virginiana*)	LC	O/FS	At least 4	—	USA	(*24*,*27*,*28*,*30*,*36*,*37*,*40*,*41*,*44*,*45*)
Rodentia	Cricetidae	Muskrat (*Ondatra zibethicus*)	LC	O	At least 1	—	USA	(*24*)
	Castoridae	Beaver (*Castor canadensis*)	LC	H	At least 1	—	USA	(*24*)
Marine mammals								
Carnivora	Phocidae	Grey seal (*Halichoerus grypus*)	LC	C	At least 40	PA-A70V, PA-V379M, HA-P152S, NP-M448V/1, NP-D455N, NA-M231, NS1-R67Q, NS1-S87T, PB2-E627K	USA, UK, Canada, Poland, Netherlands, Germany	(*24*,*27*,*28*,*30*,*31*,*37*,*40*,*41*,*44*,*45*,*55*,*56*)
	Phocidae	Harbor seal (*Phoca vitulina*)	LC	C	At least 90	PB2-S12I, PB2-E627K, PB2-D701N, PB1-P135, PB1-R211K, PB1-M523L, PB1-V527I, PB1-1728V, PB1-R480K, PB1-N375T, PB1-F2-A56V	USA, Canada, UK, Denmark, Germany	(*24*,*27*,*28*,*30*,*31*,*37*,*40*,*41*,*44*,*45*,*56*)
	Phocidae	Caspian seal (*Pusa caspica*)	EN	C	—	—	Russia	(*41*,*44*,*45*)

Order	Family	Species	Conservation status**	Diet***	Individuals affected	Mutations found****	Country	References
	Phocidae	Southern elephant seal (*Mirounga leonina*)*	LC	C	At least 2	—	Argentina	(*27*,*45*)
	Otariidae	Northern fur seal (*Callorhinus ursinus*)	VU	C	At least 1	—	Russia	(*27*,*45*)
	Otariidae	South American fur seal (*Arctocephalus australis*)	LC	C	At least 35	—	Peru, Argentina, Uruguay, Brazil	(*27*,*41*,*44*,*45*)
	Otariidae	American sea lions (*Otaria flavescens*)	LC	C	>10000	PB2-D701N, PB1-S515A, PA-R57Q, PA-T85V, PA-M861, HA-H355R, NP-Y289F, NA-A81I, NS1-D26K, PB1-L378M, PB2-Q591K	Peru, Chile, Argentina, Uruguay, Brazil	(*27*,*28*,*41*,*44*,*45*,*57*–*59*; Pardo Roa et al.†)
Cetacea	Phocoenidae	Burmeister's porpoise (*Phocoena spinipinnis*)	NT	C	At least 1	—	Chile	(*41*,*44*,*45*)
	Phocoenidae	Porpoise (*Phocoena phocoena*)	LC	C	At least 3	—	Sweden, UK, Canada	(*24*,*28*,*31*,*37*,*40*,*41*,*44*,*45*,*60*)
	Delphinidae	Chilean dolphin (*Cephalorhynchus eutropia*)	NT	C	At least 1	—	Chile	(*41*,*44*,*45*)
	Delphinidae	White-sided dolphin (*Lagenorhynchus acutus*)	LC	C	At least 1	—	Canada	(*24*,*28*,*40*,*41*,*44*,*45*)
	Delphinidae	Common dolphin (*Delphinus delphis*)	LC	C	At least 3	PB1-L378M, PA-T85V, NA-A81I, NA-S339P, M1-N87T, NS1-D26K, NS1-E60V	Peru, UK,	(*28*,*31*,*41*,*44*,*45*,*57*)
	Delphinidae	Bottlenose dolphin (*Tursiops truncatus*)	LC	C	At least 2	NA-S246N, HA-T192I	USA, Peru	(*24*,*27*,*28*,*30*,*37*,*40*,*41*,*44*,*45*; Murawski et al.‡)

* H5NX (H5 untipped).
**Conservation status based on The IUCN Red List of Threatened species: LC (Least concern); NT (Near Threatened); VU (Vulnerable); EN (Endangered); CR (Critically Endangered), NC (Non-classified).
*** Diets based on MammalBase-Database of recent mammals and the IUCN Red List of Threatened species: C (Carnivorous); FS (facultative scavenger); O (Omnivorous); H (Herbivorous).
**** To view the complete list and details of mutations found, refer to the cited bibliography.
†Pardo Roa et al., unpub. data, https://doi.org/10.1101/2023.06.30.547205.
‡Murawski et al., unpub data, https://www.researchsquare.com/article/rs-3065313/latest.

References

1. Qi X, Li X, Rider P, Fan W, Gu H, Xu L, et al. Molecular characterization of highly pathogenic H5N1 avian influenza A viruses isolated from raccoon dogs in China. PLoS One. 2009;4:e4682. PubMed https://doi.org/10.1371/journal.pone.0004682

2. Amonsin A, Songserm T, Chutinimitkul S, Jam-On R, Sae-Heng N, Pariyothorn N, et al. Genetic analysis of influenza A virus (H5N1) derived from domestic cat and dog in Thailand. Arch Virol. 2007;152:1925–33. PubMed https://doi.org/10.1007/s00705-007-1010-5

3. Songserm T, Amonsin A, Jam-on R, Sae-Heng N, Pariyothorn N, Payungporn S, et al. Fatal avian influenza A H5N1 in a dog. Emerg Infect Dis. 2006;12:1744–7. PubMed https://doi.org/10.3201/eid1211.060542

4. Jiang W, Wang S, Zhang C, Li J, Hou G, Peng C, et al. Characterization of H5N1 highly pathogenic mink influenza viruses in eastern China. Vet Microbiol. 2017;201:225–30. PubMed https://doi.org/10.1016/j.vetmic.2017.01.028

5. Reperant LA, Rimmelzwaan GF, Kuiken T. Avian influenza viruses in mammals. Rev Sci Tech. 2009;28:137–59. PubMed https://doi.org/10.20506/rst.28.1.1876

6. Kiss I, Gyarmati P, Zohari S, Ramsay KW, Metreveli G, Weiss E, et al. Molecular characterization of highly pathogenic H5N1 avian influenza viruses isolated in Sweden in 2006. Virol J. 2008;5:113. PubMed https://doi.org/10.1186/1743-422X-5-113

7. Klopfleisch R, Wolf PU, Wolf C, Harder T, Starick E, Niebuhr M, et al. Encephalitis in a stone marten (*Martes foina*) after natural infection with highly pathogenic avian influenza virus subtype H5N1. J Comp Pathol. 2007;137:155–9. PubMed https://doi.org/10.1016/j.jcpa.2007.06.001

8. Roberton SI, Bell DJ, Smith GJD, Nicholls JM, Chan KH, Nguyen DT, et al. Avian influenza H5N1 in viverrids: implications for wildlife health and conservation. Proc Biol Sci. 2006;273:1729–32. PubMed https://doi.org/10.1098/rspb.2006.3549

9. Amonsin A, Payungporn S, Theamboonlers A, Thanawongnuwech R, Suradhat S, Pariyothorn N, et al. Genetic characterization of H5N1 influenza A viruses isolated from zoo tigers in Thailand. Virology. 2006;344:480–91. PubMed https://doi.org/10.1016/j.virol.2005.08.032

10. Hu T, Zhao H, Zhang Y, Zhang W, Kong Q, Zhang Z, et al. Fatal influenza A (H5N1) virus Infection in zoo-housed tigers in Yunnan Province, China. Sci Rep. 2016;6:25845. PubMed https://doi.org/10.1038/srep25845

11. Keawcharoen J, Oraveerakul K, Kuiken T, Fouchier RA, Amonsin A, Payungporn S, et al. Avian influenza H5N1 in tigers and leopards. Emerg Infect Dis. 2004;10:2189–91. PubMed https://doi.org/10.3201/eid1012.040759

12. He S, Shi J, Qi X, Huang G, Chen H, Lu C. Lethal infection by a novel reassortant H5N1 avian influenza A virus in a zoo-housed tiger. Microbes Infect. 2015;17:54–61. PubMed https://doi.org/10.1016/j.micinf.2014.10.004

13. Thanawongnuwech R, Amonsin A, Tantilertcharoen R, Damrongwatanapokin S, Theamboonlers A, Payungporn S, et al. Probable tiger-to-tiger transmission of avian influenza H5N1. Emerg Infect Dis. 2005;11:699–701. PubMed https://doi.org/10.3201/eid1105.050007

14. Chen Q, Wang H, Zhao L, Ma L, Wang R, Lei Y, et al. First documented case of avian influenza (H5N1) virus infection in a lion. Emerg Microbes Infect. 2016;5:e125. PubMed https://doi.org/10.1038/emi.2016.127

15. Leschnik M, Weikel J, Möstl K, Revilla-Fernández S, Wodak E, Bagó Z, et al. Subclinical infection with avian influenza A (H5N1) virus in cats. Emerg Infect Dis. 2007;13:243–7. PubMed https://doi.org/10.3201/eid1302.060608

16. Songserm T, Amonsin A, Jam-on R, Sae-Heng N, Meemak N, Pariyothorn N, et al. Avian influenza H5N1 in naturally infected domestic cat. Emerg Infect Dis. 2006;12:681–3. PubMed https://doi.org/10.3201/eid1204.051396

17. Klopfleisch R, Wolf PU, Uhl W, Gerst S, Harder T, Starick E, et al. Distribution of lesions and antigen of highly pathogenic avian influenza virus A/Swan/Germany/R65/06 (H5N1) in domestic cats after presumptive infection by wild birds. Vet Pathol. 2007;44:261–8. PubMed https://doi.org/10.1354/vp.44-3-261

18. Yingst SL, Saad MD, Felt SA. Qinghai-like H5N1 from domestic cats, northern Iraq. Emerg Infect Dis. 2006;12:1295–7. PubMed https://doi.org/10.3201/eid1708.060264

19. Zhou J, Sun W, Wang J, Guo J, Yin W, Wu N, et al. Characterization of the H5N1 highly pathogenic avian influenza virus derived from wild pikas in China. J Virol. 2009;83:8957–64. PubMed https://doi.org/10.1128/JVI.00793-09

20. He L, Zhao G, Zhong L, Liu Q, Duan Z, Gu M, et al. Isolation and characterization of two H5N1 influenza viruses from swine in Jiangsu Province of China. Arch Virol. 2013;158:2531–41. PubMed https://doi.org/10.1007/s00705-013-1771-y

21. Meseko C, Globig A, Ijomanta J, Joannis T, Nwosuh C, Shamaki D, et al. Evidence of exposure of domestic pigs to highly pathogenic avian influenza H5N1 in Nigeria. Sci Rep. 2018;8:5900. PubMed https://doi.org/10.1038/s41598-018-24371-6

22. Nidom CA, Takano R, Yamada S, Sakai-Tagawa Y, Daulay S, Aswadi D, et al. Influenza A (H5N1) viruses from pigs, Indonesia. Emerg Infect Dis. 2010;16:1515–23. PubMed https://doi.org/10.3201/eid1610.100508

23. Abdel-Moneim AS, Abdel-Ghany AE, Shany SA. Isolation and characterization of highly pathogenic avian influenza virus subtype H5N1 from donkeys. J Biomed Sci. 2010;17:25. PubMed https://doi.org/10.1186/1423-0127-17-25

24. Harvey JA, Mullinax JM, Runge MC, Prosser DJ. The changing dynamics of highly pathogenic avian influenza H5N1: next steps for management and science in North America. Biol Conserv. 2023;282:110041. https://doi.org/10.1016/j.biocon.2023.110041

25. Adlhoch C, Fusaro A, Gonzales JL, Kuiken T, Marangon S, Niqueux É, et al.; European Food Safety Authority, European Centre for Disease Prevention, Control, European Union Reference Laboratory for Avian Influenza. Avian influenza overview September–December 2021. EFSA J. 2021;19:e07108. PubMed

26. Adlhoch C, Fusaro A, Gonzales JL, Kuiken T, Marangon S, Niqueux É, et al.; European Food Safety Authority; European Centre for Disease Prevention and Control; European Union Reference Laboratory for Avian Influenza. Avian influenza overview December 2021–March 2022. EFSA J. 2022;20:e07289. PubMed

27. World Organization for Animal Health. WAHIS: World Animal Health Information System. 2023 [cited 2023 Oct 30]. https://wahis.woah.org/#/home

28. Adlhoch C, Fusaro A, Gonzales JL, Kuiken T, Marangon S, Mirinaviciute G, et al.; European Food Safety Authority; European Centre for Disease Prevention and Control; European Union Reference Laboratory for Avian Influenza. Avian influenza overview December 2022–March 2023. EFSA J. 2023;21:e07917. PubMed

29. Vreman S, Kik M, Germeraad E, Heutink R, Harders F, Spierenburg M, et al. Zoonotic mutation of highly pathogenic avian influenza H5N1 virus identified in the brain of multiple wild carnivore species. Pathogens. 2023;12:168. PubMed https://doi.org/10.3390/pathogens12020168

30. US Departament of Agriculture. 2022–2023 Detections of highly pathogenic avian influenza in mammals [cited 2023 Oct 30]. https://www.aphis.usda.gov/aphis/ourfocus/animalhealth/animal-disease-information/avian/avian-influenza/hpai-2022/2022-hpai-mammals

31. United Kingdom Animal and Plant Health Agency. Confirmed findings of influenza of avian origin in non-avian wildlife [cited 2023 Oct 30].

https://www.gov.uk/government/publications/bird-flu-avian-influenza-findings-in-non-avian-wildlife/confirmed-findings-of-influenza-of-avian-origin-in-non-avian-wildlife

32. Bordes L, Vreman S, Heutink R, Roose M, Venema S, Pritz-Verschuren SBE, et al. Highly pathogenic avian influenza H5N1 virus infections in wild red foxes (*Vulpes vulpes*) show neurotropism and adaptive virus mutations. Microbiol Spectr. 2023;11:e0286722. PubMed https://doi.org/10.1128/spectrum.02867-22

33. Hiono T, Kobayashi D, Kobayashi A, Suzuki T, Satake Y, Harada R, et al. Virological, pathological, and glycovirological investigations of an Ezo red fox and a tanuki naturally infected with H5N1 high pathogenicity avian influenza viruses in Hokkaido, Japan. Virology. 2023;578:35–44. PubMed https://doi.org/10.1016/j.virol.2022.11.008

34. Rijks JM, Hesselink H, Lollinga P, Wesselman R, Prins P, Weesendorp E, et al. Highly pathogenic avian influenza A (H5N1) virus in wild red foxes, the Netherlands, 2021. Emerg Infect Dis. 2021;27:2960–2. PubMed https://doi.org/10.3201/eid2711.211281

35. Alkie TN, Cox S, Embury-Hyatt C, Stevens B, Pople N, Pybus MJ, et al. Characterization of neurotropic HPAI H5N1 viruses with novel genome constellations and mammalian adaptive mutations in free-living mesocarnivores in Canada. Emerg Microbes Infect. 2023;12:2186608. PubMed https://doi.org/10.1080/22221751.2023.2186608

36. Elsmo EJ, Wunschmann A, Beckmen KB, Broughton-Neiswanger LB, Buckles EL, Ellis J, et al. Pathology of natural infection with highly pathogenic avian influenza virus (H5N1) clade 2.3. 4.4 b in wild terrestrial mammals in the United States in 2022. Emerg Infect Dis. 2023;29:2451–60. PubMed https://doi.org/10.3201/eid2912.230464

37. Adlhoch C, Fusaro A, Gonzales JL, Kuiken T, Marangon S, Niqueux É, et al.; European Food Safety Authority; European Centre for Disease Prevention and Control; European Union Reference Laboratory for Avian Influenza. Avian influenza overview June–September 2022. EFSA J. 2022;20:e07597. PubMed

38. Chestakova IV, van der Linden A, Bellido Martin B, Caliendo V, Vuong O, Thewessen S, et al. High number of HPAI H5 virus infections and antibodies in wild carnivores in the Netherlands, 2020-2022. Emerg Microbes Infect. 2023;12:2270068. PubMed https://doi.org/10.1080/22221751.2023.2270068

39. Adlhoch C, Fusaro A, Gonzales JL, Kuiken T, Marangon S, Niqueux É, et al.; European Food Safety Authority; European Centre for Disease Prevention and Control; European Union Reference Laboratory for Avian Influenza. Avian influenza overview March–June 2022. EFSA J. 2022;20:e07415. PubMed

40. Adlhoch C, Fusaro A, Gonzales JL, Kuiken T, Marangon S, Niqueux É, et al.; European Food Safety Authority, European Centre for Disease Prevention and Control, European Union Reference Laboratory for Avian Influenza. Avian influenza overview September–December 2022. EFSA J. 2023;21:e07786. PubMed

41. Adlhoch C, Fusaro A, Gonzales JL, Kuiken T, Mirinaviciute G, Niqueux É, et al.; European Food Safety Authority, European Centre for Disease Prevention and Control, European Union Reference Laboratory for Avian Influenza. Avian influenza overview March–April 2023. EFSA J. 2023;21:e08039. PubMed

42. Cronk BD, Caserta LC, Laverack M, Gerdes RS, Hynes K, Hopf CR, et al. Infection and tissue distribution of highly pathogenic avian influenza A type H5N1 (clade 2.3.4.4b) in red fox kits (*Vulpes vulpes*). Emerg Microbes Infect. 2023;12:2249554. PubMed https://doi.org/10.1080/22221751.2023.2249554

43. Lindh E, Lounela H, Ikonen N, Kantala T, Savolainen-Kopra C, Kauppinen A, et al. Highly pathogenic avian influenza A(H5N1) virus infection on multiple fur farms in the South and Central Ostrobothnia regions of Finland, July 2023. Euro Surveill. 2023;28:2300400. PubMed https://doi.org/10.2807/1560-7917.ES.2023.28.31.2300400

44. Adlhoch C, Fusaro A, Gonzales JL, Kuiken T, Melidou A, Mirinavičiūtė G, et al.; European Food Safety Authority, European Centre for Disease Prevention and Control, European Union Reference Laboratory for Avian Influenza. Avian influenza overview April–June 2023. EFSA J. 2023;21:e08191. PubMed

45. Adlhoch C, Fusaro A, Gonzales JL, Kuiken T, Mirinavičiūtė G, Niqueux É, et al.; European Food Safety Authority; European Centre for Disease Prevention and Control; European Union Reference Laboratory for Avian Influenza. Avian influenza overview June-September 2023. EFSA J. 2023;21:e08328. PubMed

46. Tammiranta N, Isomursu M, Fusaro A, Nylund M, Nokireki T, Giussani E, et al. Highly pathogenic avian influenza A (H5N1) virus infections in wild carnivores connected to mass mortalities of pheasants in Finland. Infect Genet Evol. 2023;111:105423. PubMed https://doi.org/10.1016/j.meegid.2023.105423

47. Agüero M, Monne I, Sánchez A, Zecchin B, Fusaro A, Ruano MJ, et al. Highly pathogenic avian influenza A(H5N1) virus infection in farmed minks, Spain, October 2022. Euro Surveill. 2023;28:2300001. PubMed https://doi.org/10.2807/1560-7917.ES.2023.28.3.2300001

48. Rabalski L, Milewska A, Pohlmann A, Gackowska K, Lepionka T, Szczepaniak K, et al. Emergence and potential transmission route of avian influenza A (H5N1) virus in domestic cats in Poland, June 2023. Euro Surveill. 2023;28:2300390. PubMed https://doi.org/10.2807/1560-7917.ES.2023.28.31.2300390

49. Briand FX, Souchaud F, Pierre I, Beven V, Hirchaud E, Hérault F, et al. Highly pathogenic avian influenza A(H5N1) clade 2.3.4.4b virus in domestic Cat, France, 2022. Emerg Infect Dis. 2023;29:1696–8. PubMed https://doi.org/10.3201/eid2908.230188

50. Domańska-Blicharz K, Świętoń E, Świątalska A, Monne I, Fusaro A, Tarasiuk K, et al. Outbreak of highly pathogenic avian influenza A(H5N1) clade 2.3.4.4b virus in cats, Poland, June to July 2023. Euro Surveill. 2023;28:2300366. PubMed https://doi.org/10.2807/1560-7917.ES.2023.28.31.2300366

51. Szaluś-Jordanow O, Golke A, Dzieciątkowski T, Chrobak-Chmiel D, Rzewuska M, Czopowicz M, et al. A Fatal A/H5N1 avian influenza virus infection in a cat in Poland. Microorganisms. 2023;11:2263. PubMed https://doi.org/10.3390/microorganisms11092263

52. Sillman SJ, Drozd M, Loy D, Harris SP. Naturally occurring highly pathogenic avian influenza virus H5N1 clade 2.3.4.4b infection in three domestic cats in North America during 2023. J Comp Pathol. 2023;205:17–23. PubMed https://doi.org/10.1016/j.jcpa.2023.07.001

53. Jakobek BT, Berhane Y, Nadeau MS, Embury-Hyatt C, Lung O, Xu W, et al. Influenza A(H5N1) virus infections in 2 free-ranging black bears (*Ursus americanus*), Quebec, Canada. Emerg Infect Dis. 2023;29:2145–9. PubMed https://doi.org/10.3201/eid2910.230548

54. Kupferschmidt K. Bird flu spread between mink is a ‘warning bell’. Science. 2023;379:316–7. PubMed https://doi.org/10.1126/science.adg8342

55. Mirolo M, Pohlmann A, Ahrens AK, Kühl B, Rubio-Garcìa A, Kramer K, et al. Highly pathogenic avian influenza A virus (HPAIV) H5N1 infection in two European grey seals (*Halichoerus grypus*) with encephalitis. Emerg Microbes Infect. 2023;12:e2257810. PubMed https://doi.org/10.1080/22221751.2023.2257810

56. Puryear W, Sawatzki K, Hill N, Foss A, Stone JJ, Doughty L, et al. Highly pathogenic avian influenza A(H5N1) virus outbreak in New England seals, United States. Emerg Infect Dis. 2023;29:786–91. PubMed https://doi.org/10.3201/eid2904.221538

57. Leguia M, Garcia-Glaessner A, Muñoz-Saavedra B, Juarez D, Barrera P, Calvo-Mac C, et al. Highly pathogenic avian influenza A (H5N1) in marine mammals and seabirds in Peru. Nat Commun. 2023;14:5489. PubMed https://doi.org/10.1038/s41467-023-41182-0

58. Gamarra-Toledo V, Plaza PI, Gutiérrez R, Inga-Diaz G, Saravia-Guevara P, Pereyra-Meza O, et al. Mass mortality of marine mammals caused by highly pathogenic avian influenza A(H5N1) virus. Emerg Infect Dis. 2023;29:2553–6. PubMed https://doi.org/10.3201/eid2912.230192

59. Ulloa M, Fernández A, Ariyama N, Colom-Rivero A, Rivera C, Nuñez P, et al. Mass mortality event in South American sea lions (*Otaria flavescens*) correlated to highly pathogenic avian influenza (HPAI) H5N1 outbreak in Chile. Vet Q. 2023;43:1–10. PubMed https://doi.org/10.1080/01652176.2023.2265173

60. Thorsson E, Zohari S, Roos A, Banihashem F, Bröjer C, Neimanis A. Highly pathogenic avian influenza A(H5N1) virus in a harbor porpoise, Sweden. Emerg Infect Dis. 2023;29:852–5. PubMed https://doi.org/10.3201/eid2904.221426

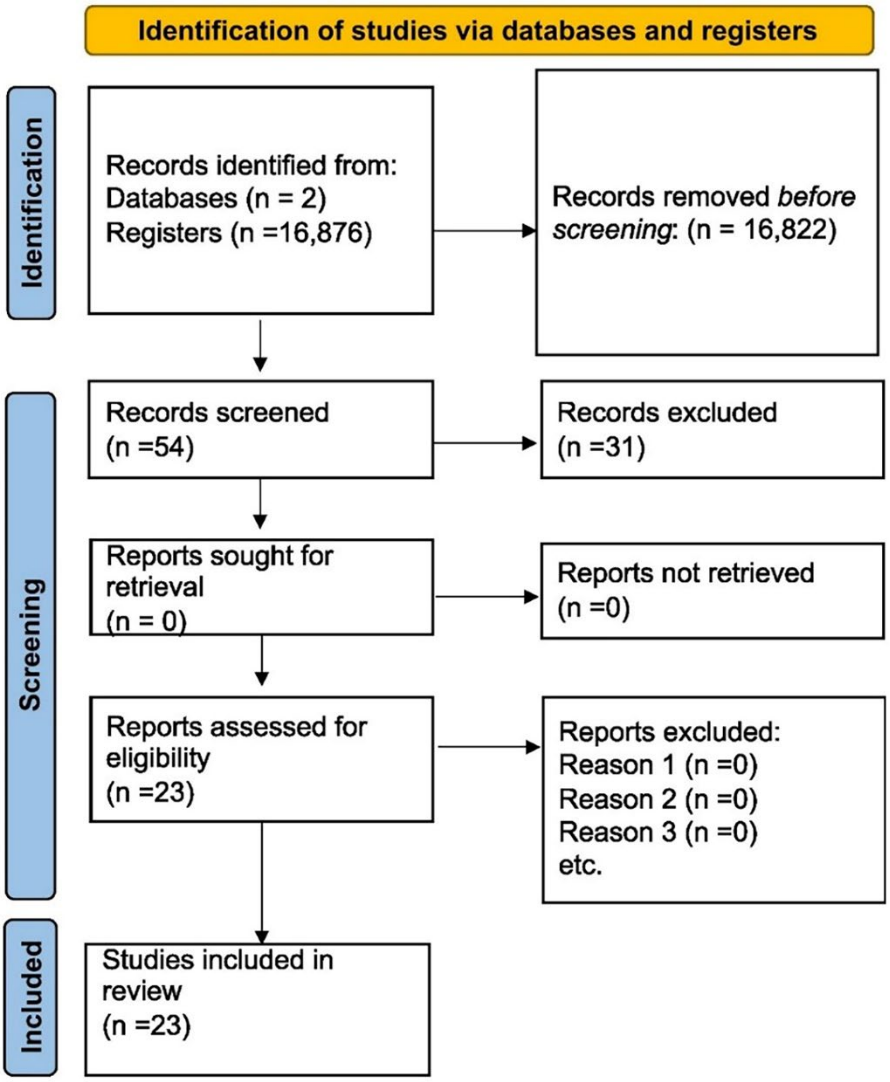

Appendix Figure 1. Prisma flow diagram with the keywords “H5N1” and “Mammals” up to 2019 (Previous waves of H5N1 infection).

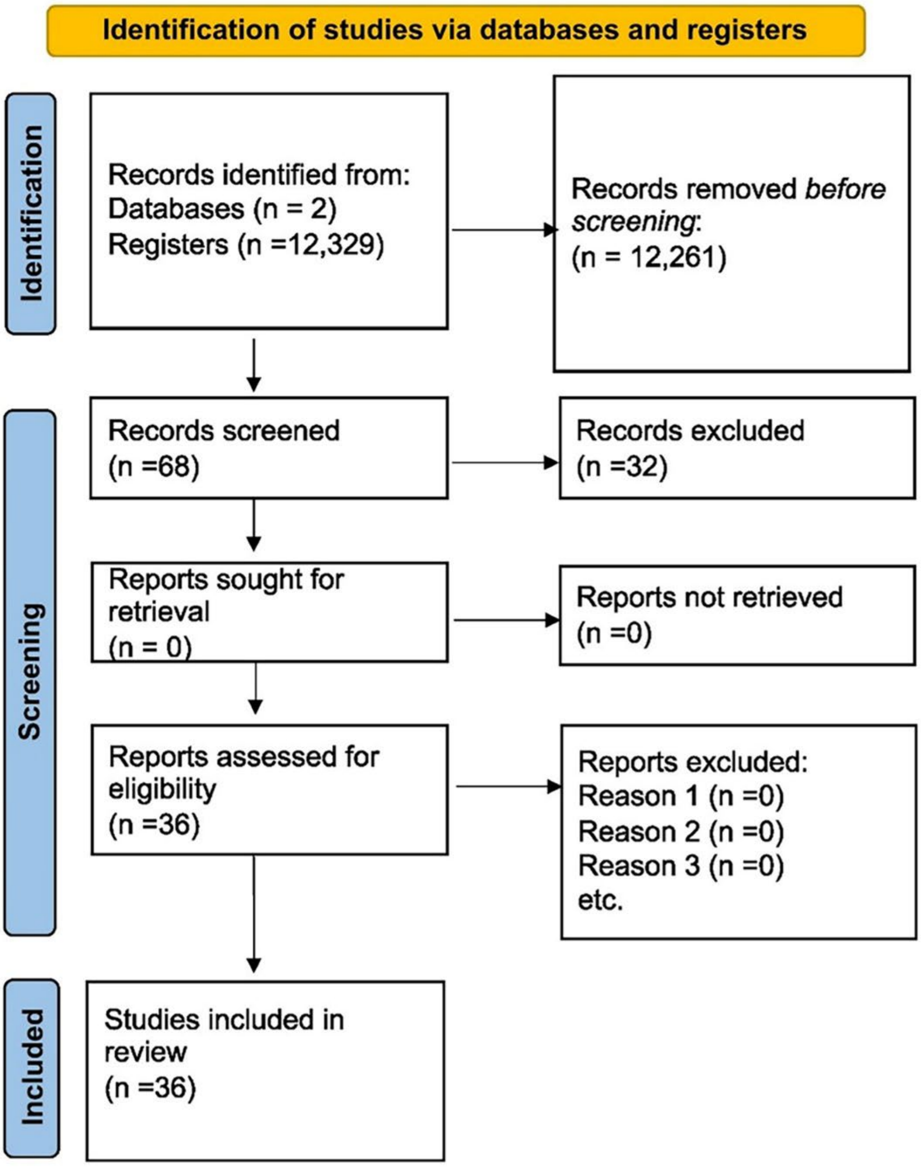

Appendix Figure 2. Prisma flow diagram with the keywords “H5N1” and “Mammals” 2020–2023 (Current panzootic 2020–2023).

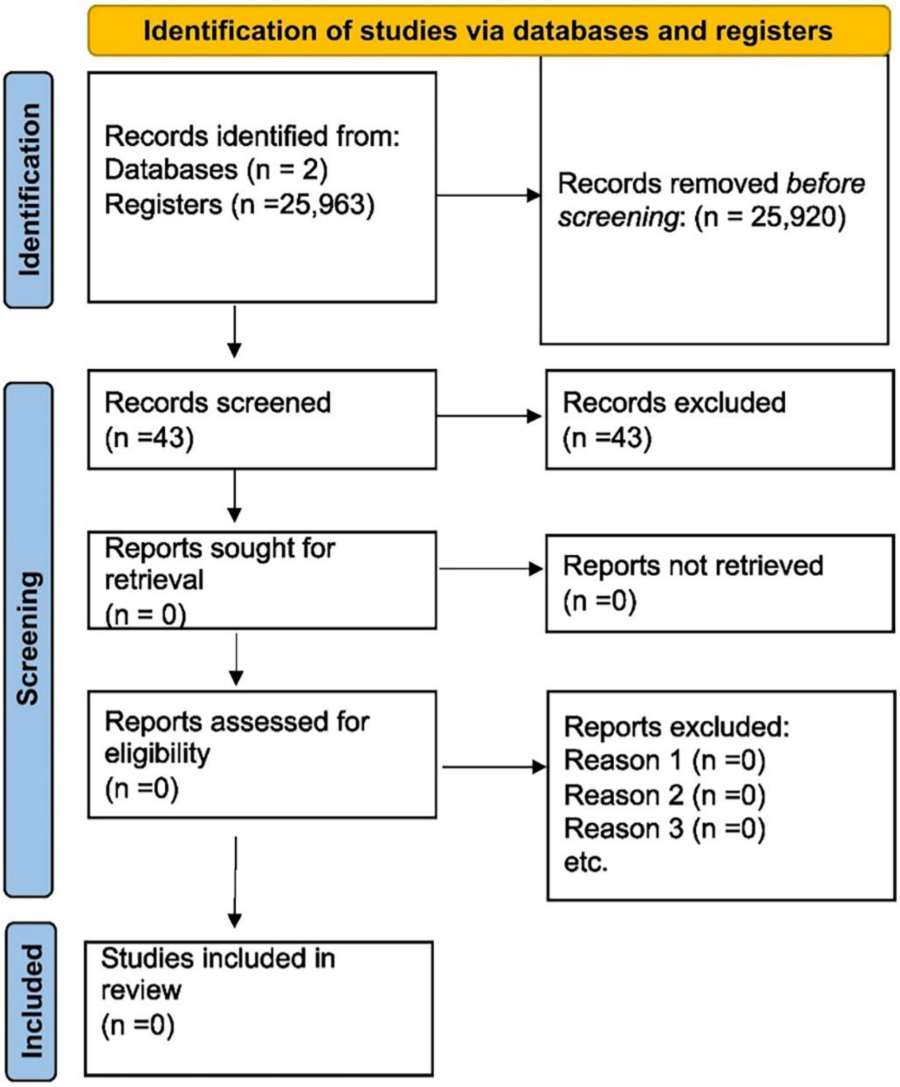

Appendix Figure 3. Prisma flow diagram with the keywords "H5N1" OR HPAI OR Highly Pathogenic Avian Influenza AND "Mammals" OR Unusual Host.
